# Rabies in an imported dog, Ontario, 2021

**DOI:** 10.14745/ccdr.v48i06a01

**Published:** 2022-06-09

**Authors:** Steven Rebellato, Mary Choi, Julian Gitelman, Felicia Ratiu, Kelly Magnusson, Brenda Armstrong, Christine Fehlner-Gardiner, Heather McClinchey, Joanne Tataryn, Maureen EC Anderson, Paul Di Salvo, Charles Gardner

**Affiliations:** 1 Simcoe Muskoka District Health Unit, Barrie, ON; 2 Dalla Lana School of Public Health, University of Toronto, Toronto, ON; 3 Canadian Food Inspection Agency, Ottawa, ON; 4 Ontario Ministry of Health, Toronto, ON; 5 Centre for Food-borne, Environmental and Zoonotic Infectious Diseases (CFEZID), Public Health Agency of Canada, Ottawa, ON; 6 Ontario Ministry of Agriculture, Food and Rural Affairs, Guelph, ON; 7 Toronto Public Health, Toronto, ON

**Keywords:** rabies, imported dog, Ontario, Canada

## Abstract

In July 2021, a dog was imported into Canada from Iran and subsequently developed clinical signs of rabies within 11 days of arrival. Following laboratory confirmation of the diagnosis of rabies, local, provincial and federal inter-agency collaboration was required to complete contact tracing to identify all persons and domestic animals that may have been exposed to the rabid dog during the potential virus shedding period. This case highlights the risks of importing animals from known canine rabies-endemic areas, identifies gaps in current dog importation policies that pose potential risk to human and animal health and prompts ongoing vigilance for this deadly disease among human and animal health partners, as well as members of the public who adopt imported dogs.

## Introduction

Requirements for importation of domestic animals into Canada are governed by the *Health of Animals Regulations* ((1)), with specific provisions for some categories of animals developed by the Canadian Food Inspection Agency (CFIA). For dogs, this may include proof of vaccination against rabies or a veterinary certificate confirming the animal has resided in a country considered free of terrestrial rabies for at least six months, though requirements differ and may be stringent, depending on age and purpose of the import (personal, assistive or commercial) ((2)). Rabies is the only disease for which Canada has specific importation requirements for dogs due to the significant public health and animal health consequences of this disease; however, the current requirements do not prevent the importation of dogs that may be incubating rabies infection in all cases.

Rabies is a viral disease that attacks the central nervous system of mammals, including humans, and is almost always fatal. Due to effective public health interventions—such as education and response to potential human exposures, effective risk assessment and management of potential domestic animal exposures, the availability of timely and reliable laboratory diagnostics, and the provision of timely rabies post-exposure prophylaxis—human cases of rabies in Canada remain rare ((3)) and Canada has been free of canine rabies since some time in the 1950s ((3)).

Nonetheless, vigilant monitoring and action by Canadian federal and provincial/territorial agencies remain crucial, particularly regarding imported dogs. The global burden of rabies is estimated to be approximately 60,000 human deaths each year, with 99% of cases associated with transmission from dogs ((4)). This is concerning given increasing human and animal movement globally, as well as low rabies vaccination rates in domestic animals in many rabies-endemic areas. In the United States, there have also been increasing reports of fraudulent or questionable rabies vaccine certificates for dogs that were imported from canine rabies-endemic countries ((5,6)).

A recent case of rabies in a dog imported from a canine rabies-endemic country to Canada illustrates some of the risks to Canadians associated with canine importation and the coordinated actions required to protect human and animal health in such cases.

## Case summary

An approximately 2-year-old mixed-breed dog (hereafter referred to as Dog 1) was imported from Iran via Europe through Toronto Pearson International Airport in Ontario, Canada on July 1, 2021. This dog was imported by a rescue organization that had prearranged its adoption by a family in Ontario.

On July 11, Dog 1 began exhibiting abnormal clinical signs, including an unspecified ocular issue, drooling and behaviour changes. The dog was assessed at a local veterinary clinic and sent home. On July 12, the dog’s clinical signs had progressed, and based on the history of importation and compatibility of the dog’s signs with rabies, the owner authorized euthanasia of the dog, and tissues were collected and submitted for rabies testing. The local public health unit (PHU) began its investigation into potential human exposures at this time, and details of the investigation are described below. The animal was confirmed to be positive for rabies based on fluorescent antibody testing performed by the CFIA rabies laboratory on July 15. Following receipt of the positive result, the local PHU expanded its investigation, which required collaboration between eight local PHUs, the provincial Ministry of Health and Ministry of Agriculture, Food and Rural Affairs, Public Health Ontario, the Public Health Agency of Canada, CFIA and the Canada Border Services Agency.

**Figure 1:**
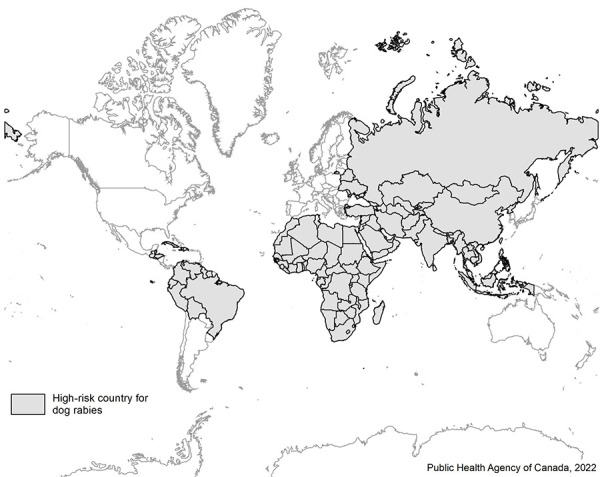
Map of high-risk countries for dog rabies^a^ ^a^ Developed by Public Health Agency of Canada. Data source (9)

Further testing by CFIA determined that the dog was infected with rabies antigenic variant IRAN-1 ((7)). Nucleotide sequencing and phylogenetic analysis corroborated the antigenic typing result and indicated the virus grouped with canine-variant viruses known to circulate in Iran and Iraq (Clade “D”) ((8)). This confirmed that the dog was infected prior to departure from Iran, which is a high-risk area for canine rabies (**Figure 1**) ((9)). Rabies is an internationally notifiable animal disease and, given that this was a novel variant to Canada, an immediate notification was submitted to the World Organisation for Animal Health by the federal government in August 2021 ((10)).

## Public health investigation

The public health investigation found that Dog 1 had travelled on international flights from Iran to Ontario via Frankfurt, Germany. When Dog 1 arrived in Ontario, it was met by a representative from the coordinating rescue organization and was transferred to a foster family for overnight lodging. On July 2, Dog 1 was transferred from the foster family to its adoptive family that subsequently introduced the dog to their extended family and friends. The dog also had contact with veterinary staff at two clinics prior to being euthanized on July 12. The dog’s adoptive family provided a veterinary health certificate from Iran that included a record of a single rabies vaccination in October 2020 using a killed rabies vaccine product.

During the investigation, a second dog (Dog 2) was identified as having travelled from Iran to Ontario in the same shipment as Dog 1, but in a separate crate. Further investigation by the CFIA and the Canada Border Services Agency yielded no evidence that the two dogs had any direct contact. Therefore, Dog 2 was not considered at increased risk of rabies exposure from Dog 1. While Dog 2 also had a record of rabies vaccination prior to importation from Iran, the dog was re-vaccinated for rabies as a precaution to ensure it was effectively vaccinated using a Canadian-licensed product, as per the requirements of the Ontario *Health Protection and Promotion Act*, Regulation 567 ((11)). No contact with any other animals (domestic or wildlife) was reported for Dog 1.

### Human contact tracing

An exposure period for contacts was established based on the defined period of communicability for rabies in domestic dogs, which is up to 10 days prior to the onset of clinical signs ((12)). Out of an abundance of caution, an exposure was defined as a person who had direct contact with Dog 1 involving a bite, scratch, or saliva exposure into a wound or mucous membrane from July 1 to July 12, 2021 (12 days).

A total of 24 individuals were identified as having contact with Dog 1 during this exposure period, of which 14 were considered exposed as described above and therefore received provincially funded post-exposure prophylaxis at an average cost of approximately CAD 2,000 per person ((13,14)). Due to the number and geographical distribution of these individuals, this required coordinated effort from multiple local PHUs and the provincial Ministry of Health. As all potential contacts were identified during this multi-jurisdictional investigation, there was no risk to the public and therefore no public risk communication was issued. High-risk contacts of Dog 1 included the foster and adoptive family members, veterinary staff, guests of the adoptive family and rescue organization personnel. No high-risk contacts were identified among airport staff. A notification was also sent to Iran via the International Health Regulations National Focal Point.

## Conclusion

This case highlights the need for ongoing vigilance for rabies among human and animal health partners, as well as members of the public who adopt imported dogs, particularly from high-risk countries. While the federal import requirement for rabies vaccination was met by the rescue organization involved, this case illustrates that this does not preclude the importation of animals incubating rabies infection and the severe consequences associated with the importation of rabid animals into Canada. Ineffective or improperly administered vaccines can also contribute to this risk, and fraudulent documentation of vaccination can be an additional compounding factor. As of July 14, 2021, the United States temporarily suspended importation of dogs from countries considered high-risk for canine rabies as a protective measure against such incidents ((15)).

Federal import requirements for dogs have been under review in Canada for several years; in May 2021 various changes were made to importation requirements for commercial dogs under eight months of age ((16)). Commercial dogs are those imported for breeding, resale and adoption end uses ((16)). This review should continue for all categories of dogs, with the aim of preventing animals infected with rabies from entering Canada. More stringent requirements for proof of vaccination with effective vaccine products (including a waiting period between vaccination and import), rabies titre testing and pre and/or post-importation quarantine requirements for dogs from designated high-risk countries could also be considered.

This incident also highlights the need for ongoing awareness among human and animal healthcare practitioners as well as public health agencies of the risks of rabies exposure from recently imported dogs ((17,18)). Public health professionals and veterinarians should strive to educate the public about the risks associated with importing animals from high-risk countries, promote consistent and timely vaccination of animals, and report any suspect imported animals to provincial and federal agencies promptly. Lastly, this incident underscores the financial and human resources costs associated with the number of local, provincial and federal agencies involved along with post-exposure prophylaxis required for high-risk contacts.
